# Planning and Developing a Symptom Diary Intervention for Breast Cancer Survivors With Concerns About Medication Brands (ENABLE Study): User-Centered Design Approach

**DOI:** 10.2196/91234

**Published:** 2026-05-26

**Authors:** Yolanda Eraso, Duncan Stewart, Ruth Edwards, Bal Nanray, Jacqueline Cochrane

**Affiliations:** 1 School of Social Sciences and Professions London Metropolitan University London, England United Kingdom; 2 Department of Pharmacy University of Wolverhampton Wolverhampton United Kingdom; 3 Patient representative Essex United Kingdom; 4 The Open University Milton Keynes, England United Kingdom

**Keywords:** intervention development, behavioral sciences, breast cancer survivors, symptom diary, qualitative methods, community pharmacy

## Abstract

**Background:**

Approximately 80% of breast cancers are estrogen receptor positive, and following initial tumor treatment, patients are prescribed hormone therapy (HT) drugs (tamoxifen, letrozole, anastrozole, and exemestane) for 5-10 years. These drugs are known to cause several side effects. Additionally, a small number of studies have identified that changing medication brands (generics) can negatively affect patients’ side effects, attitudes, and acceptance of HT. However, no effective intervention currently exists to address patients’ concerns about generic switching.

**Objective:**

This study explores whether a symptom diary appears to be an acceptable approach for patients and pharmacists to address brand concerns, and provides a detailed report of the planning and development stages of a coproduced diary intervention.

**Methods:**

This paper presents the studies conducted during the planning and development stages of the symptom diary, following the person-based approach, which combines theory-, evidence-, and person-based approaches to ensure interventions are acceptable and easy to implement. The study included the following stages: (1) the planning stage, which involved analysis of an online patient forum (n=277), interviews with patients (n=9), and interviews with pharmacists (n=7). Data triangulation was used to present an integrated set of findings. (2) The development stage, which involved a rapid scoping review of the literature on the use of diaries among patients with cancer, coproduction workshops with patients and pharmacists (n=17), pharmacist interviews (n=11), and consultation with our patient advisory group (n=5). Analysis was informed by evidence synthesis, behavior change analyses, and iterative stakeholder consultations.

**Results:**

In the intervention planning stage, patients and pharmacists perceived community pharmacies as the ideal setting for HT medication consultations. Both groups proposed using a patient diary to support awareness and attribution of drug-related symptoms. Pharmacists described patients’ requests for specific brands as difficult to manage, whereas patients reported that switching brands undermined confidence in medication-taking. In the development stage, the scoping review (n=29 papers) identified barriers and facilitators related to diary use, and workshops explored diary format and content. These findings informed the intervention planning table and the first diary prototype. Pharmacist interviews and patient feedback informed the development of 2 further prototypes. The symptom diary is a self-monitoring tool designed to enhance self-efficacy in HT medication-taking behaviors. It includes recording side effects from brands alongside physical, psychological, and environmental factors; actions taken to manage symptoms; and problem-solving and planning before a medication consultation to facilitate personalized feedback from pharmacists.

**Conclusions:**

We preliminarily developed an intervention that appears engaging, relevant, and acceptable for patients to self-monitor their symptoms. Further revisions informed by user feedback will be incorporated after testing ENABLE (Medication Brand Changes in Hormone Therapy for Breast Cancer: A Community Pharmacy Intervention Development to Improve Patients’ Adherence and Quality of Life) in community pharmacies.

**Trial Registration:**

ISRCTN Registry ISRCTN15089342; http://www.isrctn.com/ISRCTN15089342

## Introduction

Breast cancer (BC) is the most common cancer in the United Kingdom, with an estimated 56,400 new cases diagnosed in women each year [1]. Around 80% of BCs are estrogen receptor positive, meaning that, for the great majority of BC survivors, treatment does not end with surgery, radiation, or chemotherapy. Hormone therapy (HT) drugs (tamoxifen, letrozole, anastrozole, and exemestane) are prescribed for 5-10 years to reduce the risk of recurrence and mortality. These drugs are known to cause several side effects and have been consistently identified in the literature as a key factor in nonadherence [2] and poor quality of life (QoL) [3]. Adherence to HT declines by an average of 25.5% each year, and by the fifth year of treatment, adherence is estimated to range from 33.3% to 88.6%, while treatment continuation ranges from 45.2% to 87.4% [4].

In the United Kingdom, it is common practice for community pharmacies to dispense different generic brands of the same drug when patients collect their prescriptions, depending on availability and wholesalers. Currently, the British National Formulary lists 10 generic brands for tamoxifen (20 mg), 13 for letrozole, 17 for anastrozole, and 13 for exemestane, in addition to their proprietary names, when available [5]. Clinical studies on women’s experiences with HT medications have revealed that the use of generic drugs, either to replace the proprietary name (generic substitution) or to switch between generic brands (generic switching), can negatively affect patients’ side effects, attitudes, and acceptance of HT [6,7]. Among women switching tamoxifen generic brands, a small but significant proportion of patients in the United Kingdom (11%-13%) attributed severe menopausal symptoms to the brand switch, with symptoms resolving after taking a different brand. The authors suggested that patients’ perceived changes in medication side effects were due to manufacturers’ use of different excipient profiles, namely, the nonactive ingredients used to enhance manufacturing, patient acceptance, and bioavailability [6,7]. A US study on inactive ingredients concluded that this is a “poorly appreciated” area, considering the adverse reactions (allergies and irritants) that can occur in population groups taking more than 1 medication daily [8]. To date, only 1 study [9] on HT drugs has identified drug allergies as a strong predictor of adherence.

Additionally, experimental studies have shown that patients often misattribute symptoms arising from everyday activities, disease, or environmental factors as medication side effects [10], highlighting the need to assist patients in recognizing drug-related symptoms. Experiences of new side effects or worsening of existing ones after generic switching or substitution (whether excipient-related or belief-induced) can negatively affect the QoL and treatment continuation of patients with BC [6,7,11].

A growing body of evidence points to the need for interventions that provide enhanced support for patients with BC to better manage their medication [12-14]. The Royal Pharmaceutical Society acknowledges “considerable scope” for pharmacist-led interventions in cancer care, including medicines advice [15]. A systematic review on effective medicines use found that interventions that appear to improve medication adherence include medication self-monitoring and self-management programs, while promising interventions include medicines reviews, consultation with patients to resolve medication-related problems, development of a care plan, and provision of follow-up [16]. Similarly, other reviews have reported that patient medication adherence can be improved through specific community pharmacy approaches, including patient identification and enrollment, medication review, and patient assessment [17,18]. To date, no community pharmacy service or effective intervention exists to address patients’ concerns regarding HT generic switching.

This paper reports on the planning and development of a self-monitoring tool, a symptom diary, as part of the ENABLE (Medication Brand Changes in Hormone Therapy for Breast Cancer: A Community Pharmacy Intervention Development to Improve Patients’ Adherence and Quality of Life) study (NIHR206589 award; ISRCTN [International Standard Randomised Controlled Trial Number] registry ISRCTN15089342). ENABLE aims to coproduce, with patients with BC and pharmacists, an intervention to improve medication brand change (MBC) consultations in community pharmacy settings through the delivery of a patient-centered approach that could improve adherence and QoL.

The symptom diary is the patient-facing intervention component of the ENABLE study. The aims of this paper are 2-fold: (1) to explore whether a self-monitoring tool (symptom diary) appears to be an acceptable approach for patients and pharmacists to address MBC concerns; and (2) to provide a detailed account of the planning and development stages of a diary coproduced with patients, pharmacists, and stakeholders, as well as the empirical evidence and behavioral analysis that shaped its content. The latter will help explain whether a diary is an effective approach to improving consultations about HT generic brand switching, adherence, and QoL in BC survivors.

## Methods

### Study Design and Theoretical Framework

This was a multimethod qualitative intervention development study. It aligned with the person-based approach (PBA), which focuses on user-centered design to ensure that interventions are acceptable, engaging, feasible, and effective in their implementation [19]. Its theoretical approach was grounded in behavioral science and qualitative research methods to collect data on users’ perspectives at different stages of the development process. Through its combination of evidence (users’ needs and barriers and facilitators to intervention success), theory (behavioral science), and person-centered methods (qualitative research used to iteratively gather user feedback), the PBA is recognized by the Medical Research Council [20] as one of the approaches for developing complex interventions.

The intervention development methods for the symptom diary are described below in 2 main stages: planning and development. The Consolidated Criteria for Reporting Qualitative Research (COREQ) were used as the reporting guidelines for the study (Multimedia Appendix 1).

### Intervention Planning

#### Planning Stage: Identification of User Needs and Behavioral Determinants

The planning stage used triangulation of qualitative data from 3 preliminary studies to identify users’ needs, barriers and facilitators, and behavioral determinants: analysis of posts from a UK online BC forum, and interviews with patients and community pharmacists.

#### Online BC Patient Forum

Online patient forums are frequently accessed as valuable sources of health-related information, particularly for managing side effects, where patients can find supportive, experience-focused comments and advice [21]. The aim was to identify how patients with concerns about MBCs sought support for their medication, what actions they took, and what responses they received from health care professionals (HCPs). Searches were conducted in the “Hormone Therapy” section of the Breast Cancer Now forum (data accessed May 15, 2019-December 31, 2020) using the following keywords: “brand*,” “tamoxifen,” “letrozole,” “anastrozole,” “exemestane,” and specific drug brand names in use at the time. Searches covered the period from January 1, 2013, to December 31, 2020. A total of 290 women were identified, of whom 277 (95.5%) experienced new or worsening side effects after brand switches. A research assistant extracted data into a Microsoft Excel spreadsheet, including brands, year of posts, patients’ age, years of treatment, drug names, side effects, contacts with pharmacists, and interactions with other HCPs. Multiple posts by the same individual were combined into a single entry so that each individual had only 1 record.

#### Interviews With Patients With BC

A second study interviewed women with lived experience of MBCs recruited through the Breast Cancer Now charity. Participants were recruited through an invitation advertised on the charity’s research web page. Interested patients completed an online form and were contacted by email. Eligible participants were aged 18 years or older, had previously been prescribed HT, and had experience with MBCs. Nine women were included in the study and interviewed by a research assistant between July 9 and September 19, 2021. Interviews were conducted via Microsoft Teams and Zoom (Zoom Communications, Inc; n=7 interviews) or by telephone (n=2 interviews) and lasted 24-63 minutes. Participants lived in different parts of England and had a mean age of 57 years. A semistructured interview explored patients’ attitudes, needs, challenges, and preferences regarding HT brand changes and was informed by interpretative phenomenological analysis (IPA) [22].

#### Interviews With Community Pharmacists

Informed by the previous 2 studies, semistructured interviews with 7 community pharmacists located in London and the South East region were conducted by YE between March 16 and April 11, 2022. This exploratory analysis aimed to provide a more comprehensive understanding of the MBC phenomenon by triangulating our existing data with pharmacists’ perspectives on the topic. An interview guide consisting of open-ended questions explored pharmacists’ experiences with MBCs, management of consultations, the potential role of pharmacists, and the feasibility of a contract to deliver a pharmacy service for patients with BC. Saturation was sought when patterns across all 4 topics were identified. Participants were purposively selected. Eligible participants were registered pharmacists or pharmacy technicians with experience dispensing HT medication. Participants were recruited through visits to pharmacies in London and Oxford, where participant information sheets (PISs) containing a link to register interest were distributed, and through snowball sampling, whereby participants shared the PISs with contacts in the region. One pharmacist agreed to participate but did not respond to attempts to arrange the interview. No participants were known to the researchers before the interviews. Individual interviews were conducted face to face (n=3), by telephone (n=2), and via Microsoft Teams with audiovisual recording (n=2), and lasted 15-25 minutes. Participant characteristics are presented in Table S1 in Multimedia Appendix 2.

#### Analysis

Content analysis [23] was used to analyze women’s posts in the online patient forum. First, the research assistant inductively coded the posts, and the coding was audited by YE. Second, the codes were categorized according to behavioral factors using the Theoretical Domains Framework [24].

All interviews were audio recorded, transcribed verbatim, and analyzed. A patient interview guide was developed using an IPA approach [22], primarily because MBCs related to HT had not previously been qualitatively explored, and an experiential approach was sought to generate preliminary in-depth data to inform future interventions based on a PBA [19]. Further details of the study are published elsewhere [11]. Pharmacists’ interviews were analyzed using thematic analysis [25], in which codes were derived inductively by an experienced research assistant and audited by YE (25% of transcripts). The identification of themes was mapped to the study aims and discussed and agreed on by the researchers.

Finally, data triangulation across the 3 data sources was undertaken to support cross-validation and enhance the credibility of the MBC phenomenon as experienced by pharmacists and patients [26]. Findings were organized into barriers and facilitators and mapped onto the Theoretical Domains Framework [24] to analyze behavioral determinants. The synthesis is presented with an assessment of agreements and disagreements across the 3 data sets. In addition, a detailed behavioral analysis was conducted to document how the qualitative studies undertaken during the planning stage informed the development of the symptom diary intervention. Although the rich idiographic approach of the IPA study is not fully retained in this paper, data triangulation allowed us to strengthen the validity of the selected empirical evidence used in the behavioral analysis.

### Intervention Development

#### Overview

Following the planning stage, our team embarked on an intervention development process guided by the PBA [19]. A description of the methods used at each step is summarized below, following the Guidance for the Reporting of Intervention Development (GUIDED) [27] checklist for reporting intervention development (Multimedia Appendix 3).

#### Theory

The intervention followed Bandura’s social cognitive theory [28], which addresses the interplay between self-regulatory (personal) and environmental (social) determinants of health behavior. Previous systematic reviews have identified self-efficacy in medication taking as a potential predictor and modifiable factor influencing adherence and persistence with HT drugs [29,30]. Self-efficacy is a core concept in Bandura’s social cognitive theory [31] and refers to an individual’s belief or confidence in their ability to perform a specific behavior to achieve a desired outcome. Higher self-efficacy for medication taking has been significantly associated with greater HT adherence [32]. Evidence also suggests that self-efficacy is an important determinant of intentional nonadherence related to 2 other distinct behavioral components: self-efficacy in managing side effects [29] and self-efficacy in communicating with HCPs [32-34]. The latter is particularly relevant because concerns regarding MBCs increase patient-HCP communication, mainly with pharmacists and general physicians (GPs), as shown in our analysis of the online forum data below.

Self-efficacy is a precursor to self-management. In Bandura’s theory, cognitive and emotional factors (participant modeling and cognitive processing), alongside environmental factors such as verbal persuasion (social support), interact to influence self-management behaviors. Self-management in chronic conditions refers to the ability to monitor the condition (eg, side effects) and to achieve the motivation and emotional responses necessary to maintain an acceptable QoL [35]. While self-monitoring strategies, such as a symptom diary, are instrumental in increasing patients’ awareness and improving symptom management, evidence indicates that addressing a single factor to improve adherence is ineffective because of the multifaceted nature of adherence dimensions [36]. A meta-analysis on HT adherence found that only interventions involving bidirectional communication (ie, eliciting information from and providing information to patients) improved adherence compared with controls [37]. Thus, environmental factors such as social support, in the form of feedback on behaviors, represent an important source of self-efficacy for successfully managing medication-taking behaviors.

In addition, self-management programs for patients with BC are often not co-designed with patients and HCPs, which may reduce their effectiveness. Relatedly, in the United Kingdom, nonadherence to HT among ethnically minoritized patient groups is 1.48 times higher than among White British patients [38]. Patients with cancer living in deprived areas are more likely to experience delays in starting treatment (33% higher compared with those living in the least deprived areas) or delays in seeking help because they feel they would not be taken seriously [39]. These findings highlight the need for inclusive co-design approaches, as reflected in our recruitment strategies.

#### Rapid Scoping Review

Rapid scoping reviews are particularly relevant for intervention development studies because of their exploratory nature and broader scope in determining what evidence is available across heterogeneous sources [40]. For this study, the review question was: “What patient diary interventions are available to support self-monitoring behaviors in cancer survivors after active treatment?” Additional elements of interest included intervention components and data on patients’ experiences, usability, acceptability, adherence, and QoL outcomes. Studies were excluded if diaries were used solely to report symptom scales, episode frequencies (eg, pain or hot flashes), or activities performed, without promoting further user engagement (ie, not intended to facilitate patient self-management behaviors or feedback from HCPs).

Searches included systematic reviews and primary studies of any design in the following electronic databases: Cochrane Library, PubMed, CINAHL, and APA PsycINFO. Because of time constraints, searches were limited to August 1, 2004, to August 31, 2024. The search terms used for each database were adapted to derive the most meaningful results, using a combination of free-text terms, MeSH (Medical Subject Headings) terms, and subject headings. Additional key papers were identified through reference lists, targeted author searches, forward citation searching, and the authors’ expertise.

The scoping review protocol has been published [41], and the review was conducted by JC and YE. The findings were used to inform workshop discussions on the most appropriate information for patients to record about symptoms, approaches to enhance self-efficacy, and problem-solving strategies to support information exchange during medication consultations.

#### Coproduction Workshops

Coproduction methods [42] informed the workshops, with the aim of developing a symptom diary in partnership with patients with BC and pharmacists. Recruitment of patients with BC took place in London, and Breast Cancer Voices and South Asian Health Action circulated the call for contributors. Women with lived experience of MBCs and diverse sociodemographic characteristics (age, ethnicity, language, and socioeconomic status) were selected. Community pharmacy participants were recruited through Local Pharmaceutical Committee networks via RNDD North London (Community Pharmacy Research Champions) and included pharmacists, pharmacy technicians, and locums with experience dispensing HT medication. Interested individuals were asked to register via Microsoft Forms.

We planned for a workshop sample of 20 participants, equally divided between patients and pharmacists and organized into 2 mixed groups. Although there is no established guidance on workshop participant numbers [43], we considered that smaller mixed groups would better facilitate effective communication and engagement across different perspectives (patients and pharmacists). Workshop attendees included 12 patients with BC and 5 community pharmacists. A diverse group of patients with BC was purposively selected during recruitment and included participants of different ages (range 38-73 years; mean 56 years), ethnicities (8 White and 4 from minoritized ethnic groups), residential backgrounds from the most deprived areas (n=8) and least deprived areas (n=4), and 1 participant with a physical impairment. Community pharmacists’ roles included locum pharmacist (n=1), superintendent pharmacist (n=1), pharmacy managers (n=2), and pharmacist (n=1), with a range of work experience (1-35 years; mean 20.6 years), serving communities in the most deprived areas (n=3) and least deprived areas (n=2). Attendee characteristics are presented in Table S2 in Multimedia Appendix 2. A summary of findings from the rapid scoping review was circulated to participants in advance.

A face-to-face workshop was held at London Metropolitan University in October 2024 and lasted 4 hours, including lunch and coffee breaks. The meeting began with a presentation of the ENABLE study, an introduction to the National Institute for Health and Care Research key principles of coproduction [44], the role of patients and stakeholders as research contributors, and the planned activities. In all workshops, pharmacists and patients worked together in small groups with 1 facilitator (1 Breast Cancer Now nurse, or 1 patient representative, or members of the research team [DS, RE, or YE]) to encourage understanding of each other’s perspectives, experiences, and challenges. This shaped group discussions toward a more feasible and practice-focused approach to symptom diary implementation. A handbook guide was developed for each facilitator, including instructions on the aims, questions, probes, and group management. A voting system was used to complement discussions for some activities, such as a diary format and content. The session concluded with a whole-group discussion and feedback on the workshop. The workshop was audio-recorded. Additionally, 3 online workshops were held on October 25 and 30 and November 5, 2024, via Microsoft Teams with audiovisual recording, following the same structure described above. These workshops included screen sharing of presentations, tasks, and materials, with voting activities completed orally. Workshops lasted 1.5-2 hours.

#### Intervention Guiding Principles, Logic Model, and Intervention Planning Table

Based on the data triangulation conducted during the planning stage, theory-informed evidence on HT adherence interventions, the rapid scoping review, and workshop discussions, we developed the intervention guiding principles and logic model, as well as the intervention planning table (Table 3). The latter involved a behavioral analysis that first identified the mechanisms of action (MoAs) and subsequently the behavior change techniques (BCTs), based on the best available evidence reported in the Theory and Technique Tool [45], a resource for the development of theory-based interventions.

#### Patient Advisory Group

The group included 5 patients with BC with experience of MBCs from different ethnic backgrounds (South Asian, n=1; Indian, n=1; and White British, n=3), as well as BN, the patient and public involvement and engagement (PPIE) lead who chaired the group. As in previous recruitment strategies, participant selection aimed to ensure a plurality of lived experiences and demographic characteristics (see Table S3 in Multimedia Appendix 2). The initial patient advisory group (PAG) meetings were scheduled to provide feedback on the intervention components without prior involvement with the research team, in order to maximize members’ perspectives on the intervention from a fresh viewpoint. The PAG members are based in England, and meetings take place online. Breast Cancer Now and South Asian Health Action supported recruitment. Members participate in a series of scheduled meetings throughout the study and receive payment for their time in line with National Institute for Health and Care Research recommendations. The PAG reviewed diary prototype 1 and provided suggestions for prototype 2.

#### Interviews With Pharmacists

A purposive sample of community pharmacists (n=6) and pharmacists representing professional bodies (n=5), including the Royal Pharmaceutical Society, Community Pharmacy England, and National Pharmacy Association, were interviewed (see Table S3 in Multimedia Appendix 2). The PBA recommends a flexible approach to sample size during intervention design (5-10 participants, depending on study scope) [46]. The aim was to gather feedback from target users and key stakeholders on their views and practice-based experiences of the intervention design, offering new insights to complement those of the PAG, whose members had been involved in the study from the outset. Community pharmacists were recruited using the same criteria and through the organizations described above, while representatives from professional bodies were recruited through snowball sampling via a contact provided by RE. No participants were known to the researchers before the interviews, and there were no dropouts. All participants received a PIS explaining the purpose of the study. The semistructured individual interviews used a topic guide to explore views on the intervention and insights into barriers and facilitators to implementation. A synopsis of the intervention was circulated in advance, and pharmacists provided suggestions for diary prototype 2. Interviews were conducted by YE via Microsoft Teams with audiovisual recording and lasted 17-46 minutes. Interviews took place between November 28 and December 19, 2024.

#### Research Team and Reflexivity

##### Development Team

The core development team included those with expertise in community pharmacy and pharmacy education, applied health research, adherence behaviors in BC, patient involvement in research, qualitative methods, and the lived experience of BC. JC (health psychologist) has conducted research on postmastectomy lived experiences using qualitative approaches. RE (pharmacist and academic in education) and BN (patient representative) had experience with MBCs related to HT. DS (applied health researcher) has extensive experience researching pharmacy medication review services. YE (health studies) has expertise in qualitative research, with research interests in women’s health, including BC adherence, behavioral science, and intervention development. Stakeholder input included a Breast Cancer Now nurse (collaborative partner), who supports patients through the charity’s helpline and facilitated workshops, as well as reviewing diary revisions with the team. Research team meetings took place online.

##### Reflexivity

The research team, which represented a range of academic backgrounds and differing experiences and interests related to the research topic, agreed on the final analyses and interpretations presented here. Previously, we engaged in a coproduction process that addressed power imbalances through respectful and equitable relationships, while collaboratively challenging assumptions and generating and interpreting data. The iterative feedback process involving embedded groups (PAG) and external participants (pharmacists) engaged the research team in ongoing reflection on previous assumptions underlying the use of a diary.

#### Development of the Symptom Diary

We first produced a prototype of the diary, drawing on findings from the planning stage, rapid scoping review, and codevelopment workshops, which informed its format and content. The intervention planning table, alongside the intervention guiding principles and logic model, provided a framework for designing and mapping diary sections and items relevant to patient monitoring according to their potential to effect behavior change (ie, self-efficacy). Two further prototypes (2 and 3) incorporated modifications following feedback from the PAG, pharmacists, and the research team.

#### Analysis

Workshops, interviews, and meetings were recorded and transcribed verbatim by JC. Workshop materials were also collected for data analysis. Interviews with pharmacists followed a thematic analysis approach [25], including independent inductive coding by JC and YE. Data collection initially focused on capturing all participants’ preferences and suggestions, followed by consideration of disagreements and consensus based on the number of contributors supporting each view. Final decisions regarding diary design and proposed modifications were based on their likely impact on facilitating behavior change and their alignment with the study protocol (eg, suggestions to use a mobile diary were documented but were not planned within the scope of this study). Modifications were recorded in a table of changes.

### Ethical Considerations

#### Planning Stage

The studies received ethical approval from the London Metropolitan University Research Ethics Committee (approval number SSSP-4050119/SSPR 022). Written informed consent was obtained from all interviewed participants. Pharmacists received a £20 (US $27) voucher as a token of appreciation for their time. For the patient forum posts, individual consent was not obtained because of the practical challenges associated with obtaining authorization from a large online community. Authorization was granted by Breast Cancer Now forum moderators, and, following recommendations from the London Metropolitan University Research Ethics Committee, posts were paraphrased to minimize the risk of traceability through online searches.

#### Development Stage

Ethical approval was obtained from the Health Research Authority NHS (National Health Service) West Midlands-South Birmingham Research Ethics Committee (approval number 24/WM/0137) and the London Metropolitan University Research Ethics Committee (approval number SSSP-2024-025).

#### Workshops

All participants signed a consent form and received £75 (US $101) for their involvement, in line with National Institute for Health and Care Research recommendations, as well as a certificate of attendance. Pharmacists were also offered a fee for locum backfill.

#### Pharmacist Interviews

All participants provided electronic consent before the interview and were offered a £30 (US $40) voucher as a token of appreciation for their time.

## Results

### Intervention Planning

Barriers and facilitators experienced by patients and pharmacists across the 3 studies (data triangulation) are presented in Table S1 in Multimedia Appendix 4, alongside the behavioral determinants identified using the Theoretical Domains Framework [24].

### Synthesis of Three Studies

#### Beyond Patient Factors: The Role of Health Care Systems

The findings provide insight into “health care team and system factors,” including HCP interactions, drug distribution systems, drug availability (shortages), HCP beliefs about generics and side effects, and responsiveness to patients’ concerns. Health care team and system factors represent one of the multifaceted domains that the World Health Organization associates with medication nonadherence in chronic conditions [47]. Other domains include social and economic factors affecting patients, disease-related factors (disease progression and risk), therapy-related factors (treatment burden), and patient-related factors (self-efficacy and beliefs about medication) [47]. Overall, research on patients’ behaviors and psychological needs has received more scholarly attention than system factors or environmental context and resources, despite their potential to influence patients’ capability and motivation to adhere to treatment.

#### Convergences

All data sets provided evidence that some women are brand sensitive and that these experiences, alongside the search for a single or alternative brand(s), often led to fortuitous solutions depending on pharmacy stock availability, pharmacists’ efforts to procure a specific brand, or GP agreement to prescribe by manufacturer name. Community pharmacists interviewed appeared responsive to patients’ brand-seeking behaviors and attempted to secure medications within the constraints described above (see Table S2 in Multimedia Appendix 4). Most pharmacists (with one exception) believed that different brands could cause different side effects, and their explanations reflected both a biomedical model (different excipients and E numbers) and a belief in patients’ lived experiences. Although there is no scientific evidence explaining how HT brands may cause different side effects, the explanations proposed by pharmacists, particularly the possibility that excipients may affect bioavailability, were shared by patients, who often scrutinized nonactive ingredients in search of validation for their perceived differences. Patients and pharmacists viewed community pharmacies as the ideal setting for HT medication consultations. Both groups recognized the need for pharmacist training on HT drugs and patients’ medication concerns. Pharmacists also anticipated the need for clear guidelines to manage these conversations, similar to the New Medicines Service [48], to ensure consistent advice for patients. Both groups proposed the use of a patient diary to support greater awareness of side effects, drug attribution, and beliefs about medications.

#### Dissonances

Pharmacists described patients’ requests for specific brands as challenging (time-consuming and, at times, costly for the pharmacy) and difficult to manage within the constraints of National Health Service prescription services and the Drug Tariff [49]. Although patients considered cost savings to the NHS as the reason underlying generic switching, they often did not understand the NHS prescribing framework or the financial implications for pharmacies operating as businesses. Some patients in the online forum described the process as “Every prescription pick-up is an anxiety-inducing moment” [P32-PaF], particularly when they sought only 1 specific brand, whereas pharmacists viewed this behavior as frustrating and, at times, unnecessarily self-inflicted:

So at least some people, they're willing to try something if they can't get it, but I'm like, why would you want to restrict yourself?

CP5

This was compounded by patients who claimed to follow their HCP’s advice to stick to 1 brand,

So when I mentioned a side effect to the oncologist at my six-monthly review, he said: “Always stick to the same brand”...I would say [to the pharmacist]: “Can I have the AR* brand name please?” and they would say: “Well, we’re not allowed to do that”. And to be honest, I’ve been backwards and forwards so much about trying to check [this brand] that I just gave up. [Julie, interview quoted in

11] p.10

This highlights, first, that brand sensitivity is not uniquely triggered by patients and, second, a disconnect within the care team regarding how the medication supply framework operates. Patients reported that concerns about MBCs undermined their confidence in taking medication and could affect treatment continuation:

I’ve got one friend in the group..., who just stopped it altogether because she couldn’t find a brand that was supportive to her, so she stopped. But again, she was like me, she couldn’t get a discussion going about what the differences might be. [Diane, interview quoted in

11], p.9-10

We aimed to capture women’s lived experiences in our behavioral analysis (see Table S3 in Multimedia Appendix 4), scrutinizing the identified barriers, negative emotions, lack of knowledge, limited confidence in managing symptoms, and feelings of being undermined (not listened to) during conversations with HCPs, who often questioned the validity of their experiences. The latter was carefully considered in our analysis of self-monitoring behaviors (diary use) proposed by women themselves, in a way that balanced their experiences with MBCs and those of pharmacists, for whom the need for specific communication skills and guidance in managing these conversations was acknowledged. Self-monitoring through digital technologies is widely used in the prevention and treatment of chronic conditions, but it has also been associated with “individual responsibility,” shifting the burden of care onto patients [50]. Overall, women made it clear that support after discharge from the hospital was needed and that HCPs should listen to their concerns and offer better options for managing medication-related issues [11]. The research team envisaged pharmacy training and guidance as requiring alignment between existing medication consultation skills and the specificities of HT treatment in BC survivors. Further details on the online forum analysis and the patient and pharmacist interviews are available in Tables S1-S3 in Multimedia Appendix 4.

Taken together, these studies suggest that many patient interactions with community pharmacists could be construed as missed opportunities to provide care and advice for symptom management beyond a narrow focus on brand-related discussions. As it stands, system factors (wholesaler stock, medication shortages, and GP prescribing by brand name) and scientific principles (generic bioequivalence) appear to place self-management and side effect control beyond patients’ capabilities and limit the support pharmacists can provide. In summary, the 3 intervention components identified during the planning stage were informed by the preferences of patients and pharmacists and included a self-monitoring diary, a pharmacist training package, and consultation guidelines. The next section reports findings from the diary development process.

### Development of a Symptom Diary

#### Rapid Scoping Review

A total of 29 reports (including 19 studies) published between 2007 and 2024 were identified, of which 8 were from the United Kingdom. Study designs included protocols (n=4); intervention design studies using mixed methods (n=3) and qualitative methods (n=3); feasibility studies (n=4); pilot studies (n=5); randomized controlled trials (n=3); postintervention studies using qualitative (n=1), quantitative (n=1), and mixed methods approaches (n=1); systematic reviews (n=2); an overview of reviews (n=1); and a Delphi study (n=1). Most were intervention studies (n=24) involving different cancer types (eg, uterine/cervical, breast, colon, and prostate and lung cancers, among others). Diaries were used to improve physical activity and healthy diet (n=18), QoL (n=2), symptom management (n=3), and medication adherence (n=2). Diary use required completion periods ranging from 3 days to 12 months, and the format was primarily digital (web- or app-based), paper only (n=3), or both paper and digital (n=1). Most articles used diaries to record activities, including the use of scales, whereas 2 studies used a diary more as a reflective tool [51,52]. Key diary components and outcomes were organized into potential barriers and facilitators (Table 1). Although evidence regarding the effectiveness of diary interventions remains limited (only 3 randomized controlled trials [52-54]), positive outcomes were reported across heterogeneous interventions (eg, increased physical activity, improved diet, or enhanced medication adherence) using behavioral targets relevant to supporting self-efficacy in our intervention (goal setting, problem solving, and social support). Evidence regarding factors that appeared to support or hinder engagement, usability, and acceptability of diary use among patients with cancer was also considered during workshop discussions.

**Table 1:** Summary of topics, facilitators, and barriers from the rapid scoping review of the literature.

Facilitator		Barriers
**Patient enrollment/completion**		
	High intervention/study completion [52,53,55-61]	Team’s inability to reimburse lost wages or offer interactions with a health care professional outside working hours [52]. Intervention is inconvenient to everyday life/transport [58]. Participants’ unwillingness to self-manage lifestyle [52,62,63]. Anxiety about participation [55]. Ill health restricting participation [51,53,55,63-65].
**Engagement, acceptability, and usability of the diary**		
	Diaries seen as useful [54-58,61,65-69] Easy to learn and use [55,56,58,61,65,66,69,70] Cancer survivors appreciated knowledge gained about intervention target (eg, medication adherence/diet/physical activity) [51,54,58,66,69-71]	Burden, difficulty, and time commitment required for diary keeping [55,56,63]. Memory issues and fatigue [56]. Technical issues with digital diaries [55,56,61,63,66,70]. Literacy and language barriers for paper-based and electronic diaries [51,52]. Diary lacked complexity or flexibility [72].
**Interventions with a diary component and behavioral targets**		
	Focus on goal setting [51,52,55,57-59,61,63,64,67,69-71,73-75] Problem solving [51,52,54,57,58,64,67,69,71,73-76] Social support: discussion with a health care professional and feedback on plans/goals/activities [51-55,57-60,62-64,67,71,73-78]	No personalized information in automated feedback [69]. Sustained lifestyle change is hard [61]. Diary lacked interactivity [70].
**Outcomes of interventions using a diary component (self-monitoring)**		
	Increased self-management/control [53-55,66,69,70,72] Intervention aided in symptom control, supporting participants to become aware of (medications and symptoms, food quality, exercise) and plan daily routines accordingly [52-55,66,69] Improved communication with health care professionals [52,54,55,63,66,76,79] Improved intervention outcomes [52-54,60,64,65] Increased confidence [55,60,69]	A few patients found recording side effects as drawing too much attention to them [66]. Nurses found it challenging to no longer inform and advise patients in general. They were expected to facilitate patients’ own reflection [76].

#### Co-Development Workshops

Discussions were guided toward addressing key diary components based on patients’ and pharmacists’ needs and underlying behaviors identified during the planning stage, theoretical approaches related to self-efficacy in medication-taking behaviors, and findings from the scoping review identifying barriers and facilitators to diary engagement and relevant behavioral targets. The team outlined 2 broad sections for workshop discussions: (1) diary content and (2) preparation of key information for a medication consultation with a pharmacist. The discussion topics and aims are listed in Table 2.

**Table 2:** Discussion topics and their aims.

Topic for discussion	Aim
Usefulness of the diary	To explore what could be useful to record
Diary format: frequency, medium; free text and tick boxes, scales, emojis; length; and reminders	To identify components that will ensure engagement and usability
Information (hormone therapy drug common side effects and when to seek help) Symptom recording (physical and emotional) Personal circumstances and external factors (environmental and other medications) Problem solving (actions taken to control symptoms)	To gather participants’ preferences about the diary’s main content
Content summary (case scenario) Problem solving (graph and table with summary of key points)	To organize and identify key information written in the diary to use in the discussion with the pharmacist

Participants’ illustrative quotes and votes for the diary format are available in Tables S1 and S2 in Multimedia Appendix 5. At this stage, we drafted the intervention guiding principles and the logic model (see Tables S3 and S4 in Multimedia Appendix 5), which provided a framework to design and map diary sections and items for the intervention content.

#### Intervention Planning Table

The analysis reflects the barriers identified among patients with BC with concerns about MBCs, factors influencing diary engagement among patients with cancer (informed by existing evidence), and the diary components and content most likely to address these issues. The behavioral analysis identified the MoAs and BCTs most likely to be effective in facilitating behavior change, based on the best available evidence reported in the Theory and Technique Tool [45]. Table 3 presents the intervention planning table.

**Table 3:** Intervention planning table.

Barriers to target behavior	Intervention components	Mechanisms of action	BCT^a^	Content
Lack of knowledge on HT^b^ medication and side effects (PS^c^, T^d^, WS^e^, and SR^f^)	Information about potential side effects of HT drugs—supports women in identifying side effects.	Knowledge, beliefs about consequences, attitude toward the behavior, and skill (self-efficacy in deciding when to seek help)	Information about health consequences (BCT 5.1), credible source (BCT 9.1), and instruction on how to perform the behavior (BCT 4.1)	A summary list of 4 HT drugs (tamoxifen, letrozole, anastrozole, and exemestane) with common, occasional, and rare side effects provided by Cancer Research UK—Advice on when to seek professional help.
Low confidence in managing side effects from the new brand (PS, T, WS, and SR)	Support women to self-monitor their HT medication. Keeping a record of side effects from different brands: physical, psychological, and environmental factors. Record actions taken to control symptoms Raising awareness about side effects from brands (avoidance of drug misattribution)	Goals, behavioral regulation, and behavioral cuing Behavioral regulation Attitudes toward the behavior	Goal setting (behavior) (BCT 1.1), action planning (BCT 1.4), and self-monitoring of the behavior (BCT 2.3) Problem solving (BCT 1.2) Framing/reframing (BCT 13.2)	The “My Symptom Diary” section has a set of questions about brand, date of new symptoms, physical/psychological (location, severity, and impact [activities, sleep, emotions]), and environmental factors (other medications, personal circumstances, and external factors). A question prompts women to record anything done to control symptoms, and if it worked. Repeat completion, revision, and reflection on responses.
Experience disbelief about symptoms from the new brand; disregard about concerns (PS and WS)	Key summary of the diary and plans ahead of medication consultation.	Goals, motivation, beliefs about capabilities, and behavioral regulation	Goal setting (outcome) (BCT 1.3), problem solving (BCT 1.2), and reduce negative emotions (BCT 11.2)	A dedicated section in preparation for the medication consultation—summary (symptoms, environmental factors, actions to control, and medication expectations)
Lack of meaningful engagement and support from professionals regarding HT side effects (PS, T, WS, and SR)	Personalized pharmacist feedback informed by key diary summary points during a planned medication consultation (person-centered approach incorporating shared decision-making).	Goals, motivation, feedback process, environmental context and resources, and social influences	Review outcome goals (BCT 1.7), feedback on behavior (BCT 2.2), and social support (practical) (BCT 3.2)	Medication consultation starts with key summary points (diary). Medication guidelines for pharmacists (6 steps). Pharmacists and patients discuss options until a mutually agreed new approach to deal with symptoms seems acceptable. Alternatives/resolution for no decision. Notes taking, follow-up consultation.
Lack of acceptability and engagement with a diary (SR and WS): cumbersome tasks, burden (time-consuming/long), health literacy, nonnative speaker, and medium preference (paper and online)	Clear introduction on how to complete the diary Allow flexibility for diary completion (minimum 1 month to maximum 3 months) Lay language and easy to understand Discrete space for responses to questions with the option to expand Option to request from the pharmacist a paper copy or an electronic version Non-English speakers	Skills and belief about capabilities Behavioral cueing Environmental context and resources Emotion Environmental context and resources Environmental context and resources	Instruction on how to perform behavior (BCT 4.1) Action planning (BCT 1.4) and habit formation (BCT 8.3) Social support (practical) (BCT 3.2) Reduce negative emotions (BCT 11.2) Social support (practical) (BCT 3.2) Social support (practical) (BCT 3.2)	Simplicity of instructions Format is easy to navigate. Recommend writing 3 times a week. Key points: changes in brand and new/worsening of side effects; and simple, plain language, provided by the patient advisory group. The layout for responses allows for expansion (electronic version) or adding pages (paper version). Electronic version available in a free word processor; also offline to ensure accessibility. Offer translated versions of consent forms and diary in languages other than English.

^a^BCT: behavior change technique.

^b^HT: hormone therapy.

^c^PS: planning stage.

^d^T: theoretical approaches.

^e^WS: workshop.

^f^SR: scoping review.

Barriers to the target behavior identified through qualitative research informed the behavioral analysis. Some barriers, such as “experience disbelief about symptoms,” were specifically derived from data collected during the planning stage (see Table S3 in Multimedia Appendix 4) and further expanded during the development stage through workshop discussions (see Table S1 in Multimedia Appendix 5). During the workshops, participants introduced a new perspective of the diary as a form of “evidence” that could support patients’ reports of experiencing different side effects:

it might make him [pharmacist] think well, actually, you know, this does matter to her, it is important, it can happen.

PA10

There was agreement regarding the types of issues to record, except for “environmental factors/life circumstances,” for which concerns about privacy were raised by participants from minoritized ethnic groups (patients and pharmacists). This consideration was incorporated into the diary instructions as an optional component. Regarding the “key summary of the diary” prepared for the consultation (problem solving), 1 patient found documenting actions taken to control symptoms problematic because “it dilutes the fact that it's the brand that is causing this specific problem” [PA8]. These points will be important to examine in future research, as they suggest that the extent of diary completion may differ among patients who feel more certain that side effects are brand-related, which may or may not influence reflective processes before the consultation. Of particular importance were patients’ discussions about the need for pharmacist feedback to balance the burden of completing a diary, as expressed by 1 patient: “that means that you can use it [diary] to empower yourself beyond your own self patience” [PA5]. A pharmacist similarly stated, “It’ll help them [patient] and the caring team make better decisions, definitely” [CP-1]. Social support was also identified in the scoping review [66] and theoretical approaches [37]. Finally, issues related to the acceptability of and engagement with the diary were informed by findings from the scoping review and workshops, during which participants voted on the main diary features (see Table S2 in Multimedia Appendix 5).

Once the barriers to MBCs had been identified through the planning stage and workshops, they were mapped onto the MoAs, a range of behavioral theoretical constructs, including those within the Theoretical Domains Framework, that represent the processes through which specific BCTs influence behavior. The team agreed to select BCTs for which evidence of a link to a specific MoA was available in the Theory and Techniques Tool [45].

#### Symptom Diary

Prototype 1 of the symptom diary consisted of 5 sections: (1) an introduction explaining the purpose of the diary and presenting 2 scales (pain and mood) for later reference; (2) “My Symptom Diary,” containing 8 questions; (3) “Preparing Your Medication Consultation With the Community Pharmacist,” designed to summarize key points; (4) feedback on the experience of using the diary; and (5) an appendix listing side effects for each of the 4 HT drugs, categorized as common, occasional, or rare.

Details of subsequent changes to this prototype version are discussed below.

#### PAG and Pharmacist Interviews: Prototype 2

The PAG members were satisfied with the design and purpose of the diary because it allowed patients’ different experiences and perspectives on what mattered to them to be captured. They also praised the prompts encouraging consideration of environmental factors, including personal circumstances, as a valuable approach for eliciting a broad range of factors to consider.

People would perhaps think about environmental factors, so I think it's good to have all the different ones as a prompt, but it's not compulsory that they fill it in, if it doesn't apply to them. So, I thought that was good.

PG1

Regarding the diary content, members proposed the inclusion of 2 additional items to record: the date on which the medication was changed and whether the symptom had resolved. This was considered important given the possibility that medications could change while using the diary and that symptoms could fluctuate or be successfully managed by women. Members also questioned the inclusion of “fatigue” within the mood scale, as they perceived it to be a physical sensation rather than a mood-related state. We agreed to remove fatigue from the scale because the primary purpose of the diary was to facilitate women’s reflection rather than serve as a tool for quantitative data collection. Another point raised by the PAG concerned the summary of key points before the medication consultation. Patients suggested replacing “intentions” with “expectations,” given that a patient’s goals may not necessarily be fulfilled by the pharmacist, whereas “expectations” would empower women to express what they felt they needed and hoped to receive. In addition, participants suggested signposting patients to charity support before the medication consultation. Finally, 1 PAG member reviewed the entire diary for lay comprehension and made additional suggestions, which were incorporated alongside those described above.

All pharmacists were supportive of the use of symptom diaries, and many were familiar with their use for other medicines. Community pharmacists praised the diary for facilitating pharmacists’ support and increasing patient awareness.

The benefits are huge....I think it would allow patients who are not medically minded to categorise and really work out how bad it is

CP2-5

No specific comments were made regarding the diary’s set questions other than its general design: “I think make it really simple for patients” [CP2-2]. Some pharmacists interpreted the diary as giving pharmacists a more proactive role (ie, linking side effects to factors recorded for pattern observation in specific brands).

So having a symptom diary for, not just in this particular therapeutic area, but in lots of therapeutic areas is useful, to see both the symptoms and side effects and you can track and identify where those are linked to.

PH2-3

It was explained that the initial expectation was that pharmacists would not read the diary because of time constraints and privacy concerns, and that they would instead advise patients based on the patients’ presentation of key points from the diary (medication expectations valued by patients). Medicine shortages were mentioned as a challenging issue for HT drugs, echoing previous qualitative evidence. There were also comments regarding the optimal time for introducing the diary to patients (ie, at medication initiation or when concerns arise). The research team initially planned to test the intervention among those who had concerns with brands, but its suitability at medication initiation could be explored in future studies, given the reported need for information and self-management support [2,11,13,14]. Feedback was incorporated into a table of changes, with relevant quotes included for illustration (see Table S1 in Multimedia Appendix 6).

#### Research Team and Nurse Discussion: Prototype 3

A final discussion took place among the research team, the PPIE lead, and a Breast Cancer Now nurse, who received a copy of prototype 2 in advance. The team revisited components of the diary and the underpinning behavioral techniques, such as self-monitoring (confidence in recording symptoms) and problem-solving (identifying key points ahead of consultations), which were reviewed alongside feedback from the PAG and pharmacists. Many of the pharmacists’ views informed the training package (e-learning resource reported separately), in which patient-centered and shared decision-making approaches became more central to the intervention’s aim of upskilling pharmacists. Current medicine shortages were discussed as a timely opportunity to introduce the intervention, given the additional challenges associated with requesting specific brands. Specific changes related to the diary layout to ensure ease of completion and flexibility in the allocated spaces, following data from workshop contributions. Additionally, the team agreed that pharmacists would be well placed to signpost patients to charity information alongside other recommendations included in the medication consultation guide. Further details and quotes are presented in Table S1 in Multimedia Appendix 6.

Prototype 3 of the symptom diary was designed as a paper copy and in a word-processor format (to support offline completion and adaptability to systems other than Microsoft Word). The diary will be delivered by community pharmacists after training with an e-learning resource and following a medication consultation guide. Eligible patients (identified by pharmacists) are those who have previously expressed or are currently expressing concerns about MBCs. The estimated duration for completing the diary during the testing period is a minimum of 1 month and a maximum of 3 months. Prototype 3 contains the sections outlined in Textbox 1.

**Sections of prototype 3.**

1. How to use this diary

- When and what to record; presentation of pain and mood scales
- What happens next (medication consultation appointment)
- Signposting to feedback and information on drug side effects

2. My symptom diary

- Ten questions on drug name, physical and psychological symptoms, impact on daily activities, and anything done to control the symptoms
- Use of other medication(s)
- Personal circumstances and external factors
- Whether symptoms stopped

3. Preparing for your medication consultation with the pharmacist

- Summary of patients’ symptoms
- Personal life and external factors that might affect symptoms
- Actions taken to control symptoms
- Medication expectations and plans

4. Feedback notes on your experience of using the diary

- Positive and negative views

5. Common, occasional, and rare side effects of hormone therapy drugs

- Lists of tamoxifen, anastrozole, letrozole, and exemestane side effects

## Discussion

### Principal Findings

In line with the PBA, our planning stage, supported by data triangulation, was an essential first step given the scarcity of studies specifically focusing on MBCs for HT drugs. Patients’ concerns about brand changes first emerged as a potential adherence-related factor in our previous study on the impact of new guidelines extending HT from 5 to 10 years [21]. Online patient forums provide a valuable source of data, as patients perceive them as a space for sharing experiences of coping with the disease and seeking advice on managing side effects [14]. Our findings from 277 patients in the Breast Cancer Now forum, also confirmed through interviews, suggested that patients with MBC concerns encounter 2 types of HCP responses: 1 grounded in the biomedical model, which asserts that generic drugs are bioequivalent (ie, no difference exists), and 1 based on belief in patients’ lived experiences, which depends on the HCP’s sensitive approach and the patient’s ability to advocate for themselves. Data triangulation also highlighted a disconnect in HCP advice (from GPs, oncologists, and nurses) regarding the constraints of the system-level drug distribution process. These studies further deepened our understanding of the complexities inherent in conversations about brand changes. Following patients’ and pharmacists’ initial proposals regarding the use of a diary (or another method of recording side effects and brands) and a medication consultation with the pharmacist, the research team shared these ideas with PPI, stakeholders, patients, and pharmacists during the development stage. Informed by evidence on behaviors underpinning adherence to HT (self-efficacy and Social Cognitive Theory), we used qualitative research to identify MoAs, barriers and facilitators of MBCs, and candidate BCTs based on existing evidence. Drawing on the PBA for intervention development, a symptom diary was coproduced with patients and pharmacists, prioritizing acceptability and engagement with users’ preferences and, as recommended by the MRC [20], its potential implementation within the community pharmacy setting.

During the development stage, the scoping review showed that the use of diaries among patients with cancer is more effective when HCP interaction (social support) is embedded within the intervention. This aligned with a core practice standard of the General Pharmaceutical Council, which places patients’ interests and perspectives at the center of patient consultations [80]. However, these principles proved difficult to apply in practice for patients with BC who were concerned about HT generic switching. Therefore, a different approach was needed to engage patients and pharmacists in person-centered and shared decision-making practices. The diary is intended to help patients better understand symptoms and changes in brands, and to reflect on their expectations regarding the medication. This reflective process may better position patients to engage in shared decision-making consultations [81], as recommended by National Institute for Health and Care Excellence (NICE) adherence guidelines [82]. Patients’ preferences, needs, and lived experiences can “get a discussion going,” as 1 participant described it. As social support from a pharmacist appeared to be a primary motivation for diary engagement, the medication consultation strengthens the potential feasibility and acceptability of the intervention. This addresses repeated calls for clearer articulation of supported self-management within primary care provision [83], including pharmacy practice [16,34,79,84]. Furthermore, patients with BC in the England-initiated follow-up pathway have already reported a need for medication reviews [85].

Person-centered approaches focus on understanding how users engage with, use, and perceive the relevance of an intervention. This also includes HCPs such as pharmacists, who, in this study, would deliver the intervention in their practice settings. User and stakeholder engagement through coproduction and iterative feedback processes is likely to be critical for future service implementation and may support viability across diverse user groups. For example, concerns raised by ethnically minoritized patients and pharmacists about documenting personal circumstances led to making it optional for patients to decide how much personal information they wished to share in the diary. The use of lay language, readability considerations, and diary translations may further support acceptability among underserved populations. Disagreements arising during stages of the feedback process were evaluated by prioritizing the congruence of proposed modifications with the behavioral determinants and BCTs identified in our theoretical approach. Contributors’ input is transparently reported in this paper, as recommended by Slattery et al [86], making it possible to trace changes discussed by this diverse group of contributors and potential users. Further adjustments to the diary will follow completion of the testing phase in community pharmacies, informed by participant feedback.

### Limitations

Our approach was strengthened by a recruitment strategy that sought to capture a plurality of views and experiences across different sociodemographic groups. However, we acknowledge the limited inclusion of patients who do not speak English, as well as lesbian and nonbinary women. We also acknowledge that the current version of the diary could be more inclusive for women with other disabilities, including those experiencing drug-related side effects (eg, hand joint pain). In the future, a mobile version with voice-recording input could be developed alongside an offline version.

### Conclusions

This study has begun to address the need for support for patients with BC who have concerns about changes to their HT medication brands. The use of different brands causes concern for some patients, lowers QoL, and in some cases may result in poor adherence and treatment discontinuation. We reported on the planning and development of a symptom diary designed to improve patients’ self-efficacy in medication-taking and communication with pharmacists. Women perceived the diary as “empowering” because it helped them take control of their symptoms and share this information with their pharmacist, with the expectation of developing agreed-upon medication plans. Importantly, the diary is intended to complement a medication consultation with pharmacists, who will receive training and guidance as part of the ENABLE intervention. The symptom diary demonstrates the theory-, evidence-, and person-based methods used for intervention development, documenting the process used to gather qualitative evidence from patients and pharmacists, from needs identification to diary coproduction and refinement. Behavioral analysis (MoAs and BCTs) underpinned the rationale and decisions made when incorporating feedback from a diverse population group. The use of systematic intervention reporting, particularly regarding intervention components and BCT targets, will allow for further specific adjustments. It may also enhance transferability to other contexts in which generic switching is problematic for patients with other chronic conditions.

We preliminarily developed an intervention that appears engaging, relevant, and acceptable for patients to self-monitor their symptoms. Further revisions informed by user feedback will be incorporated after testing ENABLE in community pharmacies. Large-scale research will be needed to provide evidence of its impact on improving adherence and QoL.

## Appendix

Multimedia Appendix 1 The Consolidated Criteria for Reporting Qualitative Research (COREQ) checklist.

Multimedia Appendix 2 Participant characteristics. Tables S1-S3.

Multimedia Appendix 3 The Guidance for the Reporting of Intervention Development (GUIDED) checklist.

Multimedia Appendix 4 Planning stage—data analysis.

Multimedia Appendix 5 Development stage—data analysis.

Multimedia Appendix 6 Table of changes - Table S1.

## Abbreviations

BC: breast cancer
BCT: behavior change technique
COREQ: Consolidated Criteria for Reporting Qualitative Research
ENABLE: Medication Brand Changes in Hormone Therapy for Breast Cancer: A Community Pharmacy Intervention Development to Improve Patients’ Adherence and Quality of Life
GP: general physician
GUIDED: Guidance for the Reporting of Intervention Development
HCP: health care professional
HT: Hormone therapy
IPA: interpretative phenomenological analysis
ISRCTN: International Standard Randomised Controlled Trial Number
MBC: medication brand change
MeSH: Medical Subject Headings
MoA: mechanisms of action
NHS: National Health Service
NICE: National Institute for Health and Care Excellence
PAG: patient advisory group
PBA: person-based approach
PIS: participant information sheet
PPIE: patient and public involvement and engagement
QoL: quality of life

## Data Availability

Study files for the development stage are available from the OSF database: “Co-development of a symptom diary for breast cancer patients (2024-2025)” available online [87].
