# Supplementary material for: Planning and Developing a Symptom Diary Intervention for Breast Cancer Survivors With Concerns About Medication Brands (ENABLE Study): User-Centered Design Approach
Source: JMIR Cancer. 2026 May 26;12:e91234. doi: 10.2196/91234 (PMC13250491; doi:10.2196/91234)
Supplement: Multimedia Appendix 2 [file cancer_v12i1e91234_app2.docx]

**Table S1. Participant characteristics. Community pharmacist interviews (planning stage).**

| **Participant** | **Gender** | **Role** | **Yrs of work experience** | **Location** | **Pharmacy type** |
| --- | --- | --- | --- | --- | --- |
| CP1 | Female | Pharmacist | 1-10 | Oxford | Large multiple |
| CP2 | Female | Pharmacist | 20-30 | Oxford | Large multiple |
| CP3 | Female | Pharmacist | 10-20 | London | Independent |
| CP4 | Female | Pharmacist | 10-20 | Reading | Large multiple |
| CP5 | Female | Pharmacist | 10-20 | London | Independent |
| CP6 | Male | Locum | 1-10 | Oxford | Large multiple |
| CP7 | Female | Pharmacist | 20-30 | Chichester | Independent |

**Table S2. Co-development workshops. Attendees’ characteristics (development stage).**

**Patients**

| ID | Age | Ethnicity | IMD |
| --- | --- | --- | --- |
| PA1 | 60-69 | White British | 2 |
| PA2 | 50-59 | South Asian | 3 |
| PA3 | 60-69 | White British | 9 |
| PA4 | 50-59 | White British | 4 |
| PA5 | 60-69 | Black | 2 |
| PA6 | 60-69 | Indian | 6 |
| PA7 | 30-39 | Black-Caribbean | 3 |
| PA8 | 40-49 | White British | 2 |
| PA9 | 40-49 | White British | 7 |
| PA10 | 50-59 | White British | 4 |
| PA11 | 50-59 | White British | 7 |
| PA12 | 70-79 | White-other | 5 |

**Community Pharmacists**

| ID | Gender | Work experience (yrs) | Job role | Pharmacy  IMD |
| --- | --- | --- | --- | --- |
| CP-1 | Female | 10-20 | Pharmacist manager | 1 |
| CP-2 | Female | 1-10 | PR Pharmacist | 4 |
| CP-3 | Female | 30-40 | Deputy Manager | 6 |
| CP-4 | Male | 10-20 | Locum | 7 |
| #CP-5 | Female | 30-40 | Superintendent pharmacist | 3 |

**Table S3. Participant characteristics. Diary feedback (prototype 2).**

**Community Pharmacists (London)**

| ID | Gender | Years work experience | Job role | IMD |
| --- | --- | --- | --- | --- |
| CP2-1 | Female | `10-20 | Pharmacist | 4 |
| CP2-2 | Male | 30-40 | Superintendent pharmacist | 5 |
| CP2-3 | Female | 10-20 | Pharmacist owner | 2 |
| CP2-4 | Female | 40-50 | Pharmacist | 2 |
| CP2-5 | Female | 20-30 | Pharmacist | 5 |
| CP2-6 | Male | 40-50 | Pharmacist | 4 |

**Pharmacists representing professional bodies**

| ID | Gender | Area of representation | Years work experience |
| --- | --- | --- | --- |
| PH2-1 | Female | London | 20-30 |
| PH2-2 | Male | England | 20-30 |
| PH2-3 | Male | England | 1-10 |
| PH2-4 | Female | UK | 10-20 |
| PH2-5 | Female | UK | 1-10 |

**Patient Advisory Group (England)**

| ID | Ethnicity | Age range | IMD |
| --- | --- | --- | --- |
| PG1 | White British | 50-59 | 4 |
| PG2 | Indian | 60-69 | 4 |
| PG3 | White British | 50-59 | 7 |
| PG4 | White British | 60-69 | 3 |
| PG5 | South Asian | 60-69 | 3 |
