# Supplementary material for: Planning and Developing a Symptom Diary Intervention for Breast Cancer Survivors With Concerns About Medication Brands (ENABLE Study): User-Centered Design Approach
Source: JMIR Cancer. 2026 May 26;12:e91234. doi: 10.2196/91234 (PMC13250491; doi:10.2196/91234)
Supplement: Multimedia Appendix 4 [file cancer_v12i1e91234_app4.docx]

**Table S1. Data triangulation. Barriers and facilitators about HT medication brand changes, mapped to the Theoretical Domains Framework.**

| **Barriers** | Online patient forum (n=277) | Patient interviews (n=9) | Pharmacist interviews (n=7) | Theoretical Domains Framework |
| --- | --- | --- | --- | --- |
| System |  |  |  |  |
| GP exceptionally can prescribe a branded product by a manufacturer. Prescription should be by generic drug. | x | x | x | Environmental context and resources |
| Some Pa have been told they cannot request specific brands from pharmacies, unless GP prescribes it. | x | x | x | Environmental context and resources |
| Supply problems and medicines shortages affect accessibility even when brand is on prescription | x |  | x | Environmental context and resources |
| Most HCP believe there are no differences in brands (bioequivalence) | x | x | x | Environmental context and resources/ Beliefs about consequences |
| Oncologist, GP or nurse recommend patient to stick with one brand | x | x |  | Environmental context and resources / Beliefs about consequences |
| Patients |  |  |  |  |
|  |  |  |  |  |
| Pa visit/call different pharmacies asking for a specific brand and wait until brand is available | x | x |  | Intentions/ Environmental context and resources |
| Some Pa resort to a private prescription or buy medication online | x |  |  | Intentions/ Environmental context and resources |
| Pa lack information on how the supply of medication works |  |  | x | Knowledge |
| Pa experience disbelief from HCPs on their attribution of SE to changes in brands | x | x |  | Emotion/ Environmental context and resources |
| Pa believe there is no single brand that suits all patients. It’s an individual response. | x |  |  | Beliefs about consequences |
| Pa described having to fight for a brand as unfair | x | x |  | Emotion |
| Pa considered discontinuation or stopped treatment due to MBCs | x | x |  | Beliefs about consequences |
| Pharmacists |  |  |  |  |
| Focusing only on one brand is problematic (difficult to manage and harder on patient) |  |  | x | Beliefs about consequences |
| Training needs on HT drugs |  |  | x | Skills |
| Guidelines on how to manage MBCs |  |  | x | Knowledge |
| **Facilitators** | | | | |
| System |  |  |  |  |
| Pa can ask GP to add specific brand on prescription. | x | x | x | Environmental context and resources |
| Some HCP agree that different brands cause different side effects | x | x | x | Environmental context and resources/ Beliefs about consequences |
| HCPs different explanations for differences in brands (excipients, coating, manufacturer, drug absorption and metabolism) | x | x | x | Environmental context and resources/ Beliefs about consequences |
| Pharmacists can sometimes order a preferred brand, make a note on a preferred one and separate provision for a patient– depending on stock and costs | x | x | x | Environmental context and resources/ Intentions |
| Pharmacists can change generic brands if they have stock and of similar price. | x | x | x | Environmental context and resources/ Intentions |
| Patients |  |  |  |  |
| Useful to keep a diary and/or notes on brands and SE | x | x | x | Behavioural regulation |
| Identifying the brand that doesn’t agree with a patient leaves many other options to try | x | x | x | Beliefs about consequences |
| Believe pharmacies are the best setting to discuss brand changes | x | x | x | Optimism |
| Pharmacists |  |  |  |  |
| Believe a medication review of HT would support patients |  |  | x | Beliefs about consequences |
| Perceive having skills gained with existing medication reviews |  |  | x | Beliefs about capabilities |

Data include: Online Breast Cancer Now patient forum (2013-2020); Patient interviews (published paper, doi:10.3390/healthcare10122558); Pharmacist interviews (TS1 in Multimedia Appendix 1).

Pa: patients; HCP health care professional; SE side-effect

**Table S2. Themes and subthemes and quotation examples of community pharmacists (planning stage).**

| **Theme 1: Awareness and attitudes towards MBC** | |
| --- | --- |
| Experiences of patients’ concerns with MBC | ‘We do have several patients that they do want certain manufacturers [brands]’ (CP4).  ‘I tend to see [patients with brand concerns] not often, but it's not uncommon. It's not uncommon’ (CP6).  ‘Community Pharmacy is where people are very specific about the manufacturing brands of generics. Oh yeah, these brands of drugs, and also with all others, I see it more’ (CP3). |
| Perceptions of different brands causing different side effects | ‘It's no different, there's no difference in side effects because it's generic’ (CP2).  ‘Well, the thing is, I used to just think, it's the same [drug]. But then, why would so many people have these issues when their brand has changed? So now, I’d believe if a patient did complain about a particular brand and saying this is what they experience. I’d actually tend to go with it more now than I used to, to be honest. Because, you know, you see it and hear about it quite frequently now, yeah’ (CP3).  ‘It's a lot to do with the bulking agents. Some have different e-numbers and some people are allergic to sunset yellow for example, [or] they can't have anything with lactose’ (CP6). |
| **Theme 2: A difficult conversation within the NHS medicines supply system** | |
| Drug availability and pharmacists’ options | ‘About purchasing, the system that we use it's called ProScript. So usually if a doctor is prescribing a generic, PopScipt will select whichever is cost effective, and that's what we will give to the patient’ (CP2).  ‘We'd have to have a prescription because the cost [proprietary name] is way too much. We only get paid for the generic if it's the generic prescription. So typically, it all depends on that one supplier [wholesaler] and what they have in stock’ (CP6).  ‘If available at the warehouse (wholesaler), we’ll order for 6 months and keep them banded up for them [patient] specially’ (CP4). |
| Managing patients requesting only 1 brand | ‘If we don’t have what they want, we’ll offer to take the prescription elsewhere’ (CP4).  ‘Now there's a lot of medicine shortages. So, your treatment is going to be limited if it goes out of stock […] So at least some people, they're willing to try something if they can't get it, but I'm like, why would you want to restrict yourself?’ (CP5).  ‘It's a really difficult conversation to have with patients because then they feel like you're disregarding their lived experience, that you don't care and that's not true at all, [..] specially, if they have a specific brand prescribed, their life will be harder because at some point there's a good chance that they won't be able to get it. And it will cause a lot of stress because they'll be panicking, angry and upset and I've seen it so much with so many meds in general’ (CP7). |
| **Theme 3: Preferences and needs for managing MBC requests** | |
| Role of pharmacists in MBC | ‘I think it would be really good if pharmacists could get involved. If a patient wants a specific brand, then I think it'd be good if we could know which ones would cause less side effects on patients so we could take that control on ourselves’ (CP1).  ‘[For patients] it's worth keeping maybe a symptom diary, which brand you're on, um, you know just make a note of how each day is going [..] with lots of other things cause sometimes if you're feeling down in the first place, miserable or stressed, you're not going to cope with side effects very well. Which means you're going to view them as worse, and it may not have been the brand; it may have just been other life factors’ (CP7). |
| Type of service to address MBC | ‘We have something called New Medicine service. Yeah, where we give patients a phone call week 1 and then week 3, just to check up on how they get on with the medication. I think the tablets [HT] we’re discussing today should be involved in that’ (CP6).  ‘They [pharmacists] should be doing that as part of a Medication Review’ (CP7). |
| Need for guidance and training | ‘There's no guidance at the moment, nothing. I think it would be very good if we have some guidance on it’ (CP1).  ‘I think there needs to be like some training in terms of being aware of the differences in the different brands [..] I think they need to actually make it known, like issue guidance to follow’ (CP3).  ‘There should be a CPD, for example, a course where we all need to register with to provide that support […] So, I think it should be given more relevance’ (CP6). |

**Table S3. Behavioural analysis and participants’ proposed actions. Planning stage (qualitative data): Online patient forum [PaF], interviews with patients [PaI] and pharmacists [CP].**

| Barrier to target behaviour | Example quote | Target behaviour | Social Cognitive Th. | MoA | BCT |
| --- | --- | --- | --- | --- | --- |
| Lack of knowledge on HT medication and side effects | ‘I can hardly manage the stairs at the moment. And I think it might be done to this medication. Are there any generic versions with fewer side effects? What could be causing the pain in my back, joints, hips, and now my knee? I’ve also been feeling more low than usual. Thanks in advance’ (P30-PaF).  ‘So, the first change [pause] I think I did notice it, but it wasn’t too extreme, but it was when ... it was this one where it was very extreme, it’s TG*’ (Kate, PaI).  ‘[…] nobody said it to me emm ... or gave any options and you know, like if you don’t get on with this one [brand] there’s that one, or told me like to watch out for anything. Nobody said that. And I think that would have been helpful because sometimes you just panic, you know, like you think you’ve got like new symptoms and stuff’ (Liz, PaI). | Information about potential side effects of HT drugs. Support women identification of side effects. Advice on when to seek help for symptoms. | Cognitive factors (knowledge; outcome expectations)    Behavioural factors (skills) | Knowledge; Beliefs about consequences; Attitude towards the behaviour;  Skill (self-efficacy) | Information about health consequences [5.1];  Credible source [9.1]  Instruction on how to perform the behaviour [4.1] |
| Low confidence in managing SE from new brand | ‘I ended up handling it myself this time – I stopped taking Letrozole, and the side effects mostly cleared within a couple of days while I arrange for my GP to prescribe only the TG*brand or QT*brand (P41-PaF).  ‘I should be able to say: ‘No, this brand is really, I’m really not dealing with it well. I don’t want it again. Can I have ... ?’ There’s just been no dialogue about it’ (Kate, PaI). | Support women to self-monitor their HT medication.  Keeping a record of side effects from different brands.  Avoid drug misattribution. | Cognitive (self-efficacy; outcomes expectations)  Behavioural (skills) | Goals; Behavioural regulation; Behavioural cuing  Behavioural regulation  Attitudes towards the behaviour | Goal setting (behaviour) [1.1]; Action planning [1.4];  Self-monitoring of the behaviour [2.3]  Framing/ reframing [13.2] |
| Experience disbelief about symptoms from new brand; disregard about concerns | ‘I’ve asked the local pharmacist about side effects from different brands, and she said it doesn’t make a difference, but it obviously does from reading these threads. We know our bodies best’ (P147 -PaF).  ‘Every prescription pick up is an anxiety-inducing moment’ (P32-PaF).  ‘I think you’ll find most doctors, I am not sure about nurses, but most doctors will say there is no difference in brands, I mean actual brands, names, but most doctors would say there is no difference’ (Sue -PaI).  ‘I think if there would be more understanding, awareness that different brands does make a difference. Because I did feel like the pharmacist didn’t really believe me’ (Anna -PaI). | Develop plans before discussing with healthcare professionals | Behavioural (skills;  intentions)  Cognitive (self-efficacy; outcome expectations) | Goals;  Motivation;  Beliefs about capabilities; Behavioural regulation | Goal setting (outcome) [1.3] Problem solving [1.2]  Reduce negative emotions [11.2] |
| Lack of meaningful engagement and support from professionals regarding HT side effects | ‘I completely agree about working with the oncologists to get some support, but I’ve been getting mixed advice from the oncologist and different GPs I’ve spoken to. Like you said, it really comes down to weighing the pros and cons. At 39, I also find it hard to know what’s ‘normal’ or acceptable for menopause symptoms’ (P58-PaF).    ‘So when I mentioned a side effect to the oncologist at my six-monthly review, he said: “Always stick to the same brand” [ . . . ]. I would say [to the pharmacist]:“Can I have the AR* brand name please?” and they would say: “Well, we’re not allowed to do that”. And to be honest, I’ve been backwards and forwards so much about trying to check [this brand] that I just gave up’ (Julie, PaI).  ‘We have something called New Medicine service. Yeah, where we give patients a phone call week 1 and then week 3, just to check up on how they get on with the medication. I think the tablets [HT] we’re discussing today should be involved in that’ (CP6). | Prepare notes for discussion with health professional. Engage with feedback; consider next steps. | Cognitive (outcome expectations)  Environmental (social support)  Behavioural (reinforcement) | Goals;  Motivation;  Feedback process;  Environmental context and resources;  Social influences | Review outcome goals [1.7]  Feedback on Behaviour [2.2]  Social support (practical) [3.2] |

Data include: Online Breast Cancer Now patient forum (2013-2020); patient interviews (published paper, doi:10.3390/healthcare10122558); pharmacists interviews (details above).

Social Cognitive Theory: Bandura, A. A social cognitive theory of personality. In: Cervone D, Shoda Y editors. The coherence of personality: Social-cognitive bases of consistency, variability, and organization. NY: Guilford Press;1999, 185-241.

**Proposed actions by patients and pharmacists.**

| Diary use | ‘I’d keep a daily diary and note down how you feel each day just to monitor how the different brands are affecting you’ (P6-PaF).  ‘We just have to be assertive. How ready are you to try different drugs before giving up this therapy? Having some notes you can talk to the oncologist through might help to get your message across and help them to see your situation from your perspective’ (P169-PaF).  ‘I would say that anyone who is having difficulties should be advised to keep a diary and see if there is anything that they can pinpoint and it may not be the brand [ ... ] but it might be that month it was particularly hot, like it is now, or it might have been you were particularly stressed-work, kids, whatever’ (Helen, PaI).  ‘[For patients] it's worth keeping maybe a symptom diary, which brand you're on, um, you know just make a note of how each day is going […]’ (CP7). |
| --- | --- |
| Pharmacist (preferred health professional to engage) | ‘[X pharmacy] ordered me a specific brand - maybe worth speaking to the pharmacist at your local one?’ (P82-PaF).  ‘It might be worth finding a friendly local pharmacy and having a word with the pharmacist there’ (P97-PaF).  ‘The pharmacy is probably a good place actually cause they do understand what you are talking about‘ (Diane, PaI).  ‘I think it would be really good if pharmacists could get involved’ (CP1) |
