# Supplementary material for: Planning and Developing a Symptom Diary Intervention for Breast Cancer Survivors With Concerns About Medication Brands (ENABLE Study): User-Centered Design Approach
Source: JMIR Cancer. 2026 May 26;12:e91234. doi: 10.2196/91234 (PMC13250491; doi:10.2196/91234)
Supplement: Multimedia Appendix 5 [file cancer_v12i1e91234_app5.docx]

**Table S1. Co-development workshops, diary development prototype 1. Mapping contributors’ quotes to barriers to target behaviour.**

| **Barriers to target behaviour** | **Quote** |
| --- | --- |
| Lack of knowledge on HT medication and side effects | ‘I think it's helpful to have that list of symptoms because it actually gets you to think more broadly about, Oh, I didn't think that that was related to that. So, I think that gives you the option too’ (PA5). |
| Low confidence in managing SE from new brand: psychological, physical | ‘These drugs are affecting our hormones, I do think your mental state its impacted greatly anyway, so I think absolutely to include it [record in diary], yeah. There are side effects like fatigue, like low mood, all these kinds of things they're known’ (PA10). |
| Low confidence: Environmental factors | ‘I think the most important thing to note here is that environmental factors are very important to count because you know yeah, they are. We know that even though our ethnicity is elsewhere, that it takes two generations for our bodies and minds to adapt. So environmental factors influence.. it's important to put it down because problems that we have with our partners, for example, and have impact, stress... Yeah, so does have ramifications.’ (PA2).  I wouldn't want to be putting that in documenting it, yeah that document in that in any diary because yeah, because of past experiences where confidentiality has not been so great’(PA5). |
| Actions taken to control symptoms | ‘I think it empowers the patient. So, if it's yoga or it's something that they feel that they can action that they can do or have taken, and if it's worked great, and if it hasn't worked well, then it's kind of like a feedback. So, you know that you can try something else next time, but whatever it is you're recording’ (CP-5). |
| Raising awareness about side effects from brands (avoidance of drug misattribution) | ‘to just focus on what was causing what, perhaps, and to yeah and I think mainly actually is to inform me, and so that I'm aware of what I should be looking for and what might be a result of the medication, and what's nothing to do with the medication or something that might need a discussion with the pharmacist? So, I guess, it's about being informed if that makes sense?’ (PA11)  ‘I suppose that [diary] will definitely help in managing patient’s feedback. I think it's quite empowering for the patients, I imagine, because it allows them to understand better what they're experiencing. If you give a diary, it's more articulated, it's more accurate as well. It’ll help them and the caring team make better decision, definitely’ (CP-1). |
| Experience disbelief about symptoms from new brand; disregard about concerns | ‘I think what we're experiencing in the group right now is the fact that we've all had struggles and have difficulty being heard and that we feel quite strongly and quite emotional about our experiences and this group has given us an opportunity to learn to have a pop’ (PA12). |
| Lack of meaningful engagement and support from professionals regarding HT side effects | ‘When you're told at the beginning by the consultant that it's important that you stick to the same brand, it does come across a little bit concerning at times that we're sort of being given whatever [drug] is there, you know?’ (PA1).  “How are you getting on with it?” And I'll say. Bloody awful. I feel like, you know, I'm 90 years old, oh my knees, you know, my elbows kill, I could sometimes cry with my arms. And the oncologist go, ‘alright then’. Yeah, there's no point asking the question because I could just sit down and say all that’s wrong with the tablets, and the outcome is the same.’ (PA10). |
| Lack of acceptability & engagement with a diary: | ‘It can't be too complicated. Yeah, it's gonna be clear, concise and to the point because if you ask too many questions, like a big paragraph, the person's gonna think Oh, I can't be bothered to do this’ (PA6). |
| Flexibility in diary completion | ‘These drugs do have a cognitive effect on us as well, and most of us have memory impairment because of it. So, yeah, I think a daily one [entry]. I don't think I would necessarily remember if you asked me what I felt like last Wednesday’ (PA3).  See TS7 vote on diary format. |
| Discrete space for responses to questions with option to expand | ‘People would probably adhere to it better if it was short’(PA3). |
| Lay language, easy to understand | ‘You need to make it plainer English so it's more accessible to people’ (PA5). |

PA: patient; CP: community pharmacists; SE : side effects

**Table S2. Workshop attendees voting exercise. Diary development, prototype 1 (development stage).**

*Diary format Diary content*

| **Medium** | **Yes** | **No** | **Unsure** |
| --- | --- | --- | --- |
| **Paper** | 7,2P | 3,1P | 2 |
| **Online** (digital diary) | 10 | 1P |  |
| **PC/laptop** | 4, 1P | 4 | 2, 1P |
| **Mobile app** | 10, 3P | 1P |  |
| **Use voice** **recording** | 8, 2P | 1 | 2, 1P |
| **Organisation of data (other than free text)** | **Yes** | **No** | **Unsure** |
| Tick boxes | 8, 3P |  |  |
| Scales | 9, 3P |  | 1 |
| Emojis | 6 | 1 | 1 |
| **Frequency** | **Yes** | **No** |  |
| Everyday | 8, 5P |  |  |
| Weekly | 5, 1P |  |  |
| Monthly | 2 | 2, 1P |  |
| As and when needed | 4, 2P | 1P |  |
| Reminders  to complete diary | 9, 4P |  |  |
| **Length**  **(pages)** | **5 pages** | **6-8 pages** | **10-12 pages** |
|  | 12, 5P |  |  |

| **Summary of key points ahead of medication consultation** | | | |
| --- | --- | --- | --- |
|  | **Yes** | **No** | **Unsure** |
| **Content**  - information | 9,5P | 1 | 1 |
| **Format**  **-** Graph  - Table | 4,1P  1,1P | 4 |  |
| **Recording**  - Pic of notes mobile  - Writing notes (mobile or paper) | 8,3P  3,3P |  |  |
|  |  |  |  |

Notes by contributors:

a. Content description of summary of key points in graph/table was perceived as too academic. Use of user-friendly language was recommended.

b. A mobile app was mentioned by some contributors, although we made participants aware, after voting, that the research team had limited funds for this format, but we sought to gather their views for future developments.

First number corresponds to patients, second number with a ‘P’ corresponds to pharmacists.

**Table S3. ENABLE Guiding Principles**

| **Findings from literature** | **Intervention design objective** | **Key intervention features** |
| --- | --- | --- |
| Generic switching can cause new side effects in some breast cancer patients and can lead to poor adherence [6,7,11]. | Reduce patients’ concerns about generics and medication brand changes, and facilitate identification of side-effects of drugs. | i) Patients: Support patients to identify new worsening side effects and drugs attribution. Reduce concerns about drugs.  ii) Community pharmacists: Acknowledge patients’ beliefs and concerns with specific drugs. Recognise when a medication could be changed. |
| Higher self-efficacy for taking medication is associated with greater adherence in breast cancer survivors [29,32]. | Support patients to self-manage their hormone therapy medication. | A self-monitoring diary: Patients keep a record of side effects from different brands, alongside life circumstances at the time (avoid drug-side effect misattribution), and actions taken to control side-effects. |
| Patient-centred communication is a positive predictor of medication adherence in breast cancer survivors [32-34]. | Endorse a person-centred approach, with shared decision-making about medication consultations. | E-learning package for Community pharmacists: Develop empathy skills (to have a supportive conversation and listen effectively). |
| Health care professional feedback on self- monitoring interventions (diary) supports engagement, better medication adherence and quality of life in cancer survivors [37,72]. | Ability to elicit awareness and informed medication consultations. Patients and pharmacists to apply problem-solving strategies | Patients identify a summary of key points (diary) for discussion with the pharmacist.  Pharmacists use a Medication Consultation Guide with steps: listen to patients’ summary; offer unbiased information on side effects; available evidence on how to alleviate symptoms; explore options with patients, offer closure. If no agreement is achieved, offer a range of tailored alternative options for alleviating symptoms and explore other brands. |

**References**

6. Blencowe NS, Reichl C, Gahir J, Paterson I. doi: 10.1016/j.breast.2010.02.004.

7. Zeidan B, Anderson K, Peiris L, Rainsbury D, Laws S. doi: 10.1016/j.breast.2016.07.001.

11. Eraso Y, Moon Z, Steinberga I. doi: 10.3390/healthcare10122558.

29. Shelby RA, Edmond SN, Wren AA, Keefe FJ, Peppercorn JM, Marcom PK, et al. doi: 10.1007/s00520-014-2269-1.

32. Kimmick G, Edmond SN, Bosworth HB, Peppercorn J, Marcom PK, Blackwell K, et al. doi: 10.1016/j.breast.2015.06.010.

33. Liu Y, Malin JL, Diamant AL, Thind A, Maly RC. doi: 10.1007/s10549-012-2387-8.

34. Smits MAA, Mammatas LH, Schoonhoven L, Vervoort SCJM. doi: 10.1016/j.breast.2025.104510.

37. Finitsis DJ, Vose BA, Mahalak JG, Salner AL. doi: 10.1002/pon.4959.

72. Dang TH, Forkan ARM, Wickramasinghe N, Jayaraman PP, Alexander M, Burbury K, Schofield P. doi:10.2196/34833.

**Table S4. ENABLE Logic Model**

**Outputs**

**Short term outcomes**

ouomes

**Inputs activities**

A

**Mid/long term outcomes**

***** Gathering evidence (Data triangulation, review of literature, PPIE and stakeholder consultations).

***** PBA development (Behavioural analysis associated with HT medication taking).

* Breast cancer patients and pharmacists co-design intervention components. Feedback process with users.

* PAG group (feedback)

* Steering Committee (advice).

* Web-development team for E-learning resource.

* Improved adherence

* Improved QoL

* Pharmacists’ satisfaction in dealing with HT generics

* Reduced NHS costs

* A Symptom Diary for monitoring SE and psychological, physical and environmental factors. Self-manage SE through awareness and problem-solving approach before medication consultation.

* An E-learning resource to upskill pharmacists.

* A Medication Consultation Guide to support pharmacists’ delivery practice.

* Better management of MBC for patients and pharmacists.

* Accessible primary care support for patients.

* Improved control for medication taking.

* Reduced patients’ negative feelings and barriers for accessing medication.

**I n t e r v e n t I o n D e I i v e r y**

1. Pharmacists access the E-learning resource.

2. Identify patients with MBC concerns, enrol patients and offer the Symptom Diary.

NB: Dashed lines represent ultimate goals of the intervention to be explored in future studies. PBA (Person Based Approach) MBC (medication brand changes) HT (hormone therapy) PAG (patient advisory group) SE (side-effects) QoL (quality of life). [ENABLE study](https://fundingawards.nihr.ac.uk/award/NIHR206589) - NIHR206589

1. Medication consultation (10 minutes) applying the Medication Consultation Guide.

2. Record of consultation and follow-up.

1.Patients complete the diary for 1-3 months.

2. Make an appointment with pharmacist.
