# Supplementary material for: Planning and Developing a Symptom Diary Intervention for Breast Cancer Survivors With Concerns About Medication Brands (ENABLE Study): User-Centered Design Approach
Source: JMIR Cancer. 2026 May 26;12:e91234. doi: 10.2196/91234 (PMC13250491; doi:10.2196/91234)
Supplement: Multimedia Appendix 6 [file cancer_v12i1e91234_app6.docx]

**Table S1. Table of Changes**

| **PAG feedback** | **PAG quote** | **Diary modification & justification** | **Implementation time: N=now / F = future** |
| --- | --- | --- | --- |
| Simple, plain language | ‘For the layperson, consider that the average reading age in the UK, sadly, is 9 years of age. Whatever we convey is as simple as possible as can be and certainly not using convoluted language’ (PG5). | One patient provided amendments to the text in the diary to check for comprehension. | N |
| Record the date of brand switch and when symptoms stopped | ‘I thought it'd be useful to record the date you switched medication.  [...] ‘taking medication for a long period of time and they keep switching brands, it can be difficult to remember’.  […]  ‘Is it continuing [symptom] or has it stopped because so much depends on the type of symptom you have? (PG1). | Added 2 questions, a) date of 1^st^ symptom, and b) ‘Did the symptom stop? Provide date’. | N |
| Mood scale (delete fatigue & activity) | ‘Fatigue and activity I would say it's a bodily thing not mood’ (PG3). | Scale was taken from Profile of Mood States. PAG felt strongly fatigue should be part of symptom description or a new scale, but not mood. We deleted it from scale and decided not to add a new scale to make it less bothersome to patients | N |
| Use ‘expectation’ instead of ‘intention’ | ‘Expectation perhaps empowers the woman to kind of write what she feels that she needs and what she would perhaps like to get. Intention may not be fulfilled by the pharmacist’ (PG5). | PAG agreed that expectation was fine provided it does result in an outcome of the task of writing a diary. Change of words from ‘your intentions, to ‘Your medication expectations & plans’ | N |
| Suggest patients to contact Breast Cancer Now for new ideas | ‘You could maybe signpost them to something like [charity] to talk to some of their advisors around what they suggest, because we've heard some great examples today of how that organisation has been really helpful’ (PG4). | We agreed we will explore with charities whether this was possible for them to provide and where best to use their support. | N – explore with charities |
| **Pharmacists’ feedback** | **Pharmacist quote** | **Diary modification & justification** | **Implementation time: N=now / F = future** |
| Symptom diary as a tracking tool for pharmacists | ‘So having symptom diaries for again not just in this particular therapeutic area, but in lots of therapeutic areas is useful we use it a lot in asthma […] it's useful to see both the symptoms side effects that people are having and then you can sort of track and start to identify where those are kind of linked and maybe attributable to the medication versus something else’ (PH2-3). | It was explained to pharmacists that it was not expected for them to review the diary (and track symptoms and patterns), but to advice based on patients’ summary.  No modification for diary, but for e-learning resource (describe elsewhere). | N |
| Timing for using the diary: some pharmacies consider when this was appropriate, most thought not at the start of treatment to avoid ‘expectation’ of side effects. | ‘For me it would only be patients where the brand [they want] was out of stock that I would have to have that conversation, I wouldn't need to have this conversation like for the others, do you know what I mean? In my pharmacy, this wouldn't happen because they don't have symptoms or significant ones because they're on the brand that they want – these patients that I have, who are brand specific absolutely would not be taking it if they couldn't get this brand’ (CP2-5). | We made this clearer in the diary (by highlighting the word after) that the diary was for women to record new symptoms or worsening of new symptoms after a change in brands. | N |
| **Research team, BCN nurse discussion** | **Quote** | **Diary modification & justification** | **Implementation time: N=now / F = future** |
| Layout | ‘The text doesn’t fit’ words being cut in half’ (Nurse).  ‘Swap the columns and rows around. The difference that would make isn't just presentational (keeping the font size), but it would be easier to track week by week’ (Team researcher).  ‘I think if it was a table going right across with four cells and a heading for each, there will be more room’ (Team researcher). | After exploring the layout suggested, we swapped the order of rows as it produced the effect anticipated.  The graph figure to complete the summary of key points was changed for a table. | N |
| Charity engagement | ‘I'm sure there's not obviously a problem with the actual signpost, it's where it sits, I guess.  Because we say we don't give advice, I think it would be more along the lines of for more information, if you want to explore more. So, people know that at any part of the process they can contact us’ (BCN nurse). | We relocate the signposting of charity support to the Medication consultation guide. | N |

PAG: Patient Advisory group (PG); Pharmacists: community pharmacists (CP) and pharmacists representing professional bodies (PH); BCN nurse: Breast Cancer Now nurse.
